# Amebic Liver Abscess in HIV-infected Patients, Republic of Korea

**DOI:** 10.3201/eid1303.060894

**Published:** 2007-03

**Authors:** Wan Beom Park, Pyoeng Gyun Choe, Jae Hyun, Sung-Han Kim, Ji Hwan Bang, Hong Bin Kim, Nam Joong Kim, Myoung-don Oh, Kang Won Choe

**Affiliations:** * Seoul National University College of Medicine, Seoul, Republic of Korea

**Keywords:** HIV, amebiasis, liver abscess, letter

**To the Editor:** Amebic liver abscess (ALA) is the most common extraintestinal complication of amebic infection. Although loss of cellular immunity is thought to play a role in infection by the pathogen, whether HIV infection is also a risk factor for invasive amebiasis is controversial (*1*–*3*). ALA in HIV-infected patients has not been well characterized, although several case series have been reported (*2*,*4*). We report the role of HIV infection status in ALA in an area where ALA is not endemic and the clinical features of ALA in HIV-infected patients.

All patients with ALA at Seoul National University Hospital (SNUH) from January 1990 through December 2005 were identified; some have been previously reported (*5*). SNUH is a 1,600-bed, university-affiliated teaching hospital and the largest referral center for HIV/AIDS in the Republic of Korea. The diagnostic criteria for ALA were radiologic evidence of intrahepatic abscess, trophozoites of *Entamoeba histolytica* in fluid aspirated from an abscess, or absence of bacteria and fungi in aspirated fluid and a titer ≥128 in an indirect hemagglutination assay (IHA) for *E*. *histolytica*.

Of 31 patients with ALA at SNUH from 1990 through 2005, 10 (32%) were HIV positive. The proportion of HIV-infected patients among patients with ALA increased significantly with time (linear-by-linear association, p<0.001) (Figure). Of 10 patients from 1998 through 2005, 8 (80%) were HIV positive. Except for 2 patients with a history of travel to an ALA-endemic area, 88% of the patients were HIV positive.

**Figure:**
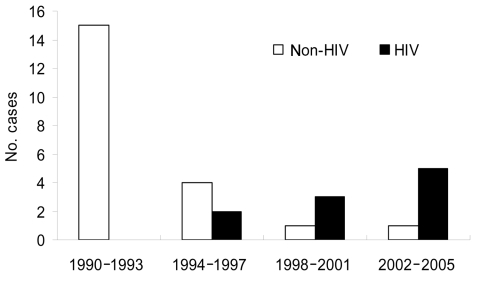
Number of cases of amebic liver abscess in patients with and without HIV infection at Seoul National University Hospital, Republic of Korea, 1990–2005.

Median age of the 10 HIV-positive patients with ALA was 34.5 years (range 29–54 years); all patients were male. Four had a homosexual orientation, 4 had a heterosexual orientation, and 2 had an unknown sexual orientation. Fever (100%) was the most common symptom, and abdominal tenderness (90%) and diarrhea (50%) were frequently observed. Median leukocyte count was 9,000/mm^3^ (range 3,410–16,700/mm^3^), and median CD4 cell count was 279/mm^3^ (range 40–370/mm^3^). Eight patients had abscesses in the right lobe of the liver and 2 had abscesses in both lobes; 8 patients had 1 abscess and 2 had multiple abscesses. Median size of abscesses was 7.25 cm (range 3–12 cm). In 5 patients, pleural effusion was observed in chest radiographs. IHA titer was ≥128 in 10 patients and ≥512 in 8 patients. Median days to defervescence was 2 (range 1–5 days). In 2 patients, perforation of the abscess into the abdominal cavity was a complication. No patients died or had relapses.

Early in the AIDS pandemic, some studies reported that the prevalence of invasive amebiasis was not increased in patients with HIV infection (*1*,*6*). However, recent reports of ALA associated with HIV infection have increased. Studies in Taiwan demonstrated that invasive amebiasis, including ALA, is on the increase in HIV-infected patients in disease- endemic areas (*2*,*7*).

Amebiasis was previously an endemic disease in the Republic of Korea. The positive rate for cysts of *E*. *histolytica*/*E*. *dispar* in the general population was 10% in the 1960s (*5*). However, with improvements in sanitation, this rate decreased to 0.5% in 1993 and to nearly 0% in 2004 (*8*). The present study showed that ALA in association with HIV infection is increasing in the Republic of Korea, and that ALA in HIV-negative patients has greatly decreased. In a study in the United States, 38% of patients with ALA with no history of travel to a disease-endemic area were HIV positive (*3*). These results support the view that ALA is an emerging parasite infection in HIV-infected patients in non–disease-endemic areas, as well as in disease-endemic areas.

Immune suppression is an important risk factor for ALA. In animal studies, immune suppression after thymectomy or splenectomy results in an increased incidence of ALA (*9*). Suppressed cellular immunity caused by use of steroids and malnourishment predispose to fatal amebiasis (*10*). In the present study, 90% of patients had CD4 cell counts <350/mm^3^, which implies that immune suppression by HIV infection may be another risk factor for ALA.

Only 2 patients with pyogenic liver abscess were observed during the study. In these patients, pyogenic liver abscess was diagnosed, despite negative cultures for bacteria and fungi, because trophozoites of *E*. *histolytica* were not demonstrated in aspirated pus, and results of IHA for amebiasis were negative. However, we cannot exclude amebic liver abscess in these 2 patients because IHA test results can be negative in HIV-infected patients (*2*).

This study suggests that ALA is an emerging parasite infection in HIV-infected patients even in areas where the disease is not endemic. ALA should be considered in HIV-infected patients with space-occupying lesions in the liver, and HIV screening is strongly recommended in patients in areas where ALA is not endemic, especially those with no history of travel to a disease-endemic area.
